# Posttranslational Modifications of HIV-1 Integrase by Various Cellular Proteins during Viral Replication

**DOI:** 10.3390/v5071787

**Published:** 2013-07-16

**Authors:** Yingfeng Zheng, Xiaojian Yao

**Affiliations:** Laboratory of Molecular Human Retrovirology, Department of Medical Microbiology, University of Manitoba, 508-730 William Avenue, Winnipeg, R3E 0W3, Canada; E-Mail: umzhen28@cc.umanitoba.ca

**Keywords:** HIV, integrase, posttranslational modification, ubiquitination, SUMOylation, acetylation, phosphorylation

## Abstract

HIV-1 integrase (IN) is a key viral enzyme during HIV-1 replication that catalyzes the insertion of viral DNA into the host genome. Recent studies have provided important insights into the multiple posttranslational modifications (PTMs) of IN (e.g., ubiquitination, SUMOylation, acetylation and phosphorylation), which regulate its multifaceted functions. A number of host cellular proteins, including Lens Epithelium‑derived Growth factor (LEDGF/p75), p300 and Ku70 have been shown to interact with IN and be involved in the PTM process of IN, either facilitating or counteracting the IN PTMs. Although previous studies have revealed much about the important roles of IN PTMs, how IN functions are fine-tuned by these PTMs under the physiological setting still needs to be determined. Here, we review the advances in the understanding of the mechanisms and roles of multiple IN PTMs.

## 1. Introduction

Proteins translated from mRNA undergo various posttranslational modifications (PTMs) [1]. In general, 15 of 20 amino acids (aa) can be modified in different ways (e.g., phosphorylation, methylation, acetylation, ADP-ribosylation, glycosylation, ubiquitination, SUMOylation and lipid additions) [1]. These modifications increase the functional diversity of the proteome and play crucial roles in the normal cell biology and disease outcomes. Microbial pathogenesis is accompanied by various PTMs occurring in proteins, both pathogen proteins as well as the proteins of host origin [2]. These modifications either promote the survival and propagation of pathogens or overwhelm the host defense systems [2,3,4]. During HIV-1 infection, several viral proteins, including proteases, envelope glycoprotein, Tat and integrase (IN) undergo different types of PTMs, which greatly impact the different steps of viral replication [5,6,7,8,9,10,11,12]. 

**Figure 1:**
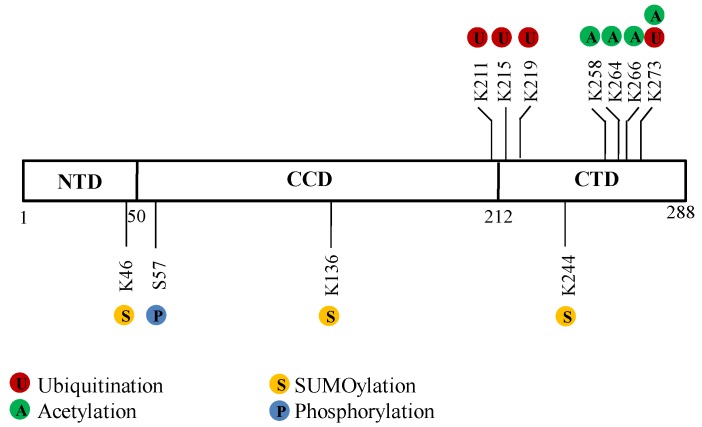
Sites of posttranslational modifications (PTMs) on HIV-1 integrase (IN). The schematic representation of the 288-amino acid domain structure of HIV-1 IN showing the amino acids subject to various PTMs, including ubiquitination, SUMOylation, acetylation and phosphorylation. Abbreviations: N-terminal domain (NTD); catalytic core domain (CCD); C-terminal domain (CTD).

**Figure 2:**
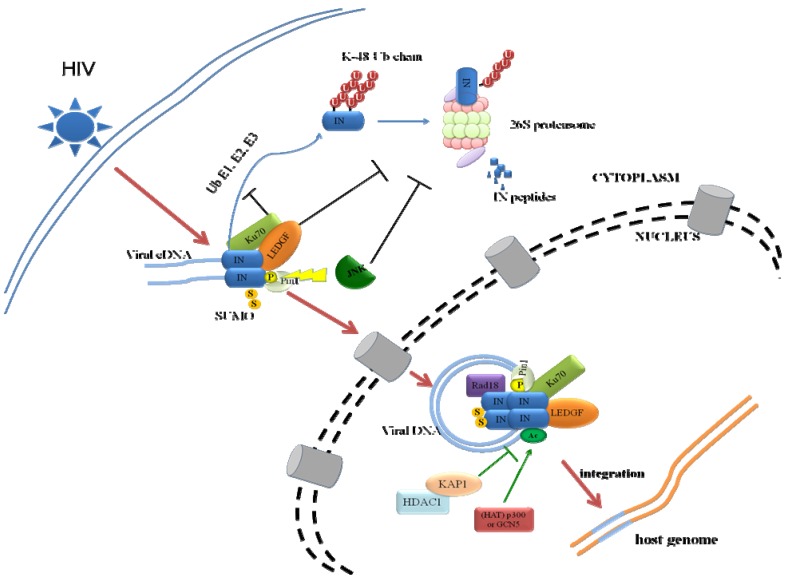
Model for regulation of IN by various PTMs. The interactions of IN with LEDGF/p75, hRad18 and Ku70 prevent IN from K48-linked Ub proteasome degradation pathway. LEDGF/p75 prevents IN from degradation through the formation of IN‑LEDGF/p75 complex which stabilizes IN. Ku70 reduces total Ub level in the cell and specifically decreases Ub modification of IN through their protein-protein interaction. Protection of IN from proteasomal degradation by hRad18 is independent of its N-end rule, but its molecular mechanism involved is still unclear. Pin1 binds phosphorylated IN which is catalyzed by JNK, leading to conformational change and prolonged half-life of IN. Therefore, phosphorylation of IN antagonizes ubiquitination of IN. The fate of SUMOylated IN is currently unknown. Both phosphorylation and SUMOylation of IN seem to occur after reverse transcription but before integration. IN is acetylated by two HATs p300 and GCN5. Acetylation of IN enhances IN/DNA binding affinity and integration. KAP1 binds and deacetylates IN by recruiting HDAC1. For clarity, other cellular cofactors of IN and viral proteins have been omitted in the figure. Abbreviations: integrase (IN); post-translational modification (PTM); Lens Epithelium-derived Growth factor (LEDGF); human Rad18 (hRad18); Ubiquitin (Ub); c-Jun N-terminal kinase (JNK); histone acetyltransferase (HAT); histone deacetylase 1 (HDAC1); small ubiquitin-like modifier (SUMO).

HIV-1 IN is encoded by the HIV *pol* gene. IN is first synthesized as part of the Gag-Pol polyprotein, in which Pol is cleaved into three viral enzymes, reverse transcriptase, protease and IN, during the maturation step. IN is a 288-aa, 32-kD viral protein that contains three distinct structural domains, the N-terminal zinc-binding domain (NTD, residues 1–50), the central catalytic core domain (CCD) and the C-terminal domain (CTD, residues 212–288) (Figure 1) [13]. IN is a pleiotropic protein that affects different steps throughout the viral life cycle, including reverse transcription, nuclear import of the preintegration complex (PIC), integration and post-integration steps, such as viral protein expression, transcription, packaging and processing [14,15,16,17]. As a single viral protein, HIV-1 IN interacts with numerous cellular cofactors in a temporally and spatially specific manner and exhibits multifunctional properties, the interactions of which are tightly regulated by different PTMs events. IN has been shown to be modified by four PTMs: ubiquitination, SUMOylation, acetylation and phosphorylation [5,10,11,12,18]. IN-interacting proteins either facilitate or counteract IN PTMs (Table 1 and Figure 2). For instance, p300 acetylates IN, whereas Ku70 reduces the ubiquitination level of IN [5,12]. PTMs on IN play central roles in the functions of IN and viral replication, affecting the stability and conformational structure of IN, DNA binding of IN, integration and infection of the virus [5,10,11,12,18]. Despite an increasing understanding of the IN PTMs, the full functions of these modifications and how these modifications coordinate with each other following viral infection are still unknown. The focus of this review is to summarize the various IN PTMs modulated by host proteins and the related functions during viral infection. 

**Table 1:** Human proteins implicated in the PTMs of IN.

Human proteins	PTM type	Interaction sites in IN	Mechanisms	Reference
LEDGF/p75	Ubiquitination	W131, W132, 161–170	Inhibits Ub proteasome degradation	[19,20]
Ku70	Ubiquitination	230–288	Inhibits ubiquitination by decreasing cellular ubiquitin level and deubiquitinates IN through their interaction	[12]
hRad18	Ubiquitination	NA	Inhibits ubiquitination	[21]
VBP1	Ubiquitination	43–195	Promotes ubiquitination by targeting IN to E3 ligase	[22]
Cul2/VHL ligase	Ubiquitination	NA	Acts as Ub E3 ligase and promotes ubiquitination	[22]
p300	Acetylation	264–288	Acetylates IN, increases IN affinity to DNA, and promotes integration	[5]
GCN5	Acetylation	244–288	Acetylates IN, enhances enzymatic activity of IN	[23]
KAP1	Acetylation	NA	Binds and deacetylates IN by recruiting HDAC1, reduces integration	[24]
JNK	Phosphorylation	NA	Phosphorylates IN	[11]
Pin1	Phosphorylation	NA	Binds phosphorylated IN, leading to conformational changes and stabilization of IN from ubiquitination	[11]

## 2. Ubiquitination of HIV-1 IN

### 2.1. What Is the Signal for Degradation: N-Degron and Lys-48-Linked Polyubiquitination Chain

Ubiquitination is a reversible PTM, in which ubiquitin (Ub) conjugates to the substrate proteins through covalent binding between the C-terminal Gly of Ub and the Lys residue of substrates via a cascade of enzyme reactions. Three classes of enzymes are involved with conjugating Ub to the substrates: Ub-activating enzyme (E1), Ub-conjugating enzyme (E2) and Ub-ligase (E3). Following the attachment of the first Ub, a polyubiquitination chain is formed. Polyubiquitinated proteins are then recognized by the 26S proteasome, which catalyzes the degradation of the ubiquitinated protein and the recycling of ubiquitin. 

IN is known to be a metabolically unstable protein. In the absence of other viral proteins, IN presents as a short-lived protein in transfected cells and has increased expression with proteasome inhibitor treatment [18]. The N-terminal Phe of IN has been suggested to be the degradation signal or N-degron [18]. After proteolytic cleavage from the Gag-pol precursor, HIV-1 IN bears natural Phe in its N-terminus, which belongs to a type 2 N-degron (bulky hydrophobic, including Phe, Leu, Trp, Tyr, and Ile) [25,26]. This previous study revealed that the N-terminal residue of IN (N-Phe) accounts for its instability, and replacing Phe with a stabilizing residue (Met, Val, and Gly) is able to stabilize IN [18]. A similar finding was obtained in another independent study [26]. Therefore, the degradation signal for IN fits into the general N-end rule pathway, which has been observed in mammals, plants and bacteria [27]. 

Protein substrates can be mono-ubiquitinated or poly-ubiquitinated, both of which confer distinct properties to the substrate proteins. Ub has 7 internal Lys (K6, K11, K27, K29, K33, K48, and K63). All of these Lys can bind to the C-terminal Gly of the preceding Ub, forming different Lys-Gly linked polyubiquitination chains. The K48-linked polyubiquitination chain (conventional chain) is the main signal for 26S proteasome degradation. Nonconventional chains, such as K63-linked polyubiquitination chain, have nonproteolytic functions, including DNA repair and stress response [28,29,30]. However, studies have also revealed that all of the polyubiquitination chains can participate in either proteolytic or non-proteolytic processes in different protein contexts [31]. One study indicated that IN presented the highest expression level when it was cotransfected with Ub mutant K48R, which is defective for the formation of K48-linked polyubiquitination chain [29], whereas IN is less stable in the presence of wild-type and Ub mutant K63R [12]. This study provides direct evidence that IN is degraded through the K48-linked polyubiquitination proteasome pathway. 

In searching for which Lys residue(s) of IN is linked with the K48-polyubiquitination chain and subject to proteasome degradation, a study by Mousnier* et al.* discovered that replacing Lys 211, 215, 219, or 273 with Arg in IN slowed down its degradation, suggesting that these Lys might be at least some of the targets of Ub [22] (Figure 1). When Lys 211, 215, and 219 of IN were all mutated into Arg in the virus, a 60% reduction of HIV infectivity was observed [22]. However, further studies are still needed to verify whether the reduction of viral infectivity is associated with the prolonged degradation of these IN mutations in the context of viral infection. 

### 2.2. What Is the Ub E3 Ligase for IN?

Ub E3 ligase, which determines the substrate specificity, catalyzes the covalent binding of Ub‑substrate and Ub-Ub multimer formation through isopeptide bond [30]. There are two types of E3 ligases differentiated by their unique domains: HECT (homologous to E6-AP carboxy-terminus) and RING (Really Interesting New Gene) fingers [30]. Two RING-finger domain-containing E3 ligase complexes have been identified as E3 ligases for IN. By using knockout and knockdown technique, an early study suggested that mammalian UBR1 (ubiquitin ligase N-recognin 1), UBR2, and UBR4, as part of the cellular E3 ligase complex, accounted for the recognition and degradation of IN by the Ub proteasome system (UPS) [26]. This type of E3 ligase recognizes substrates through direct binding to the N-terminal residue of protein substrates, including Phe in IN [25]. Mousnier* et al.* presented a distinct IN degradation model in which both the prefoldin chaperone subunit von Hippel-Lindau binding protein 1 (VBP1) and the Cul2/von Hippel–Lindau protein (VHL) ligase interacted with IN and coordinately mediated the polyubiquitination and proteasome degradation of IN [22]. It remains unanswered whether these two E3 ligases cooperatively or independently regulate the proteasome degradation of IN.

In addition to these two RING-finger containing E3 ligases, a HECT-type E3 ligase, Huwe1, (HECT, UBA and WWE domain containing 1) has also been shown to interact with both IN and the IN domain of the Gag-Pol precursor protein. However, Huwe1 does not appear to be involved in the modulation of the ubiquitination and proteasome degradation of IN and the Gag-Pol precursor protein in the overexpression system [32]. Similarly, the E3 ligase human Rad18 (hRad18), which possesses a RING-finger domain, does not degrade IN but rather it binds and protects IN through an as yet unknown mechanism [21]. Thus, IN appears to utilize different components of the host UPS for its own benefit during HIV-1 infection. 

### 2.3. How Does IN Escape Host Degradation?

Although HIV-1 IN is subject to rapid degradation in the absence of other viral proteins, it is clear that IN stays stable during a large portion of the viral life cycle. To date, a few cellular proteins have been shown to interact with IN and shield it from the host proteasome degradation pathway. Lens epithelium-derived growth factor (LEDGF/p75) was the first cellular protein reported to have a protective role on IN stability [19]. LEDGF/p75 was discovered as an IN-interacting protein using a chemical cross-linking and mass spectrometry approach in 2003 [33]. Since then, multiple roles of LEDGF/p75 in HIV-1 integration and infection have been revealed. The protective role of LEDGF is supported by a few lines of evidence. For example, IN is less stable in LEDGF-knockdown cells. Artificial overexpression of LEDGF/p75 outside of the viral life cycle increases IN expression [19]. It has been highlighted that LEDGF/p75 protects IN from proteasome degradation, dependent on their interaction but not the nuclear or cytoplasmic localization of both proteins [19]. A subsequent study undertaken by Mousnier* et al.* suggested that the protective role of LEDGF/p75 might be the result of masking the interaction site for VBP1 binding within IN, which bridges IN with Ub E3 ligase Cul2/VHL [22]. It should be noted that all these findings are derived from the data from overexpression of IN or LEDGF-knockdown. The real scenario of the protection from proteasomal degradation by LEDGF/75 under HIV-1 infection still remains a challenging problem.

In addition, two DNA repair proteins, hRad18 and Ku70, have been reported to interact and stabilize HIV-1 IN. hRad18 contains a RING-finger domain shared by E3 ubiquitin ligase, and it has been shown to target proliferating cell nuclear antigen (PCNA) for monoubiquitination [34,35,36]. However, hRad18 was observed to stabilize IN independent of its N-end rule in an overexpression system [21]. Nonetheless, the mechanisms by which hRad18 prevents IN from proteasomal degradation are still unknown. Another IN-interacting protein, Ku70, which also protects IN from proteasomal degradation, is one of the components of the non-homologous end joining (NHEJ) pathway [37,38]. The NHEJ pathway has versatile functions during HIV-1 infection, such as promoting apoptosis of infected cells, association with the PIC, viral cDNA circularization, retroviral DNA integration and suppression of HIV transcription [39,40,41]. The study found that Ku70 is incorporated into the HIV-1 virion, and both virus-associated and cell-associated Ku70 are critical for the stability of IN during viral replication [12]. This protection of IN from proteasome degradation might be ascribed to the deubiquitinating activity of Ku70 [12,42]. In addition to its general effect on the host UPS, Ku70 specifically reduces the ubiquitination level of IN through their interaction [12]. More functional studies are still needed to investigate the exact roles of cellular proteins including (but not limited to) LEDGF/p75, hRad18 and Ku70 in the regulation of IN metabolism during HIV-1 infection. 

### 2.4. Where Does Degradation Take Place — Cytoplasm *versus* Nuclei?

IN is synthesized and incorporated into virions as part of Gag-Pol polyproteins, and during maturation, IN is cleaved from the Gag-Pol polyprotein by a viral protease inside the virion. After cleavage, the N-terminal Phe of IN is exposed. The degradation of IN does not appear to take place inside the virion after budding, despite the fact that ubiquitin is indeed incorporated into the HIV-1 virion [43,44]. Rather, several lines of evidence indicate that the degradation of IN occurs in both the cytoplasm and nucleus of infected cells. The notion that IN is degraded in the cytoplasm is supported by the facts that cytoplasmic IN has a faster degradation rate than the nuclear portion, and that the cytoplasmic fraction of IN is stabilized in the presence of a proteasome inhibitor [19,45]. During viral infection, HIV PIC sheds most of its capsid proteins after entry [46], rendering its components, including HIV-1 IN, accessible to the host environment. Alternatively, it has been proposed that degradation of IN might take place inside the nucleus after the completion of integration but before viral transcription [22]. This model is supported by the indirect evidence that the cellular proteins VBP1 and Cul2/VHL Ub ligase complex together contribute to the degradation of IN and function in the transition of integration to transcription [22]. To support this notion, it has been suggested that after completion of strand transfer, IN must be degraded from the integration intermediate to allow the cellular DNA repair pathway to fill in the gap and proceed into transcription [18]. 

IN is protected by multiple cellular cofactors, such as LEDGF/p75, Ku70 and hRad18, against the host UPS to catalyze 3' processing in the cytoplasm and 5' strand transfer close to the host chromatin. However, IN is inevitably exposed to the host UPS, which is ubiquitous in the nucleus and cytoplasm of all cells. Currently, how IN interacts with various cellular proteins, in terms of protection or destruction in a timely regulated manner, still remains largely unknown. 

## 3. SUMOylation of HIV-1 Integrase

In addition to ubiquitination, IN also undergoes small ubiquitin-like modifier (SUMO) modification [10]. Similar to the ubiquitination cascade, SUMOylation is catalyzed by SUMO activating enzyme E1 (a heterodimer of Aos1 and Uba2), which is the unique E2 conjugating enzyme Ubc9, and a number of different E3 ligases, such as protein inhibitor of activated STAT (PIAS) and RAN binding protein 2 (RanBP2) [47]. This whole process is reversed by specific isopeptidases or deSUMOylases. The outcomes for SUMO modification vary greatly from substrate to substrate, including protein stability, cytosolic-nuclear translocation, antagonizing other PTMs and transcriptional regulation [48]. SUMO proteins are ~10 kD in size, and there are four subtypes (SUMO 1-4) in mammals [48]. SUMO 1, 2 and 3 are ubiquitous in the cells and share a globular ubiquitin-like shape, whereas SUMO4 is only detected in certain tissues and organs [48,49]. Whereas SUMO2 and SUMO3 are 96% identical to each other, SUMO1 only shares 45% amino acid identity with SUMO2/3. SUMOs covalently conjugate to protein substrates through a canonical four-aa SUMOylation consensus site, ψ-K-x-D/E (where ψ is a hydrophobic aa and x is any aa). 

SUMOylation of IN is newly identified and its function is as yet poorly understood. A study found that IN contains three ψ-K-x-D/E motifs, and it is SUMOylated at three Lys residues, K46, K136 and K244 [10] (Figure 1). However, SUMOylation of IN is not completely abolished with all these key Lys residues mutated, implying the presence of other SUMOylation sites. Furthermore, SUMOs have three major isoforms (SUMO1-3), all of which have been shown to modify IN in both* in vitro* and *in vivo* studies. However, it is still unknown which SUMO subtype(s) preferentially target IN and whether IN is mono-SUMOylated or poly-SUMOylated. It has been suggested that SUMOylation of IN might occur between reverse transcription and integration during viral replication, while it exerts no direct effect on its enzymatic activity or IN-LEDGF/p75 interaction. Therefore, the exact functions of IN SUMOylation in HIV-1 infection still await further characterization [10]. Of note, conjugated SUMO proteins provide a platform to recruit SUMO-interacting motif (SIM)-containing cofactors through non-covalent binding. For example, the SIMs of human TRIM5α binding to SUMO-conjugated capsid protein restricts murine leukemia virus infection [50], and RanBP2 SIM mediates its binding with the complex of RanGAP1/SUMO1 and Ubc9 [51]. Interestingly, IN has been shown to bind SUMO1/2 in a co-immunoprecipitation assay and a yeast two-hybrid assay [52], whereas some IN‑interacting proteins, such as LEDGF/p75, Ku70, p300, are SUMOylated [41,53,54,55]. Therefore, it is intriguing to consider that IN bears one or more SIMs that mediate IN/SUMO interaction and binding to SUMO-conjugated cofactors, thereby modulating the activities and functions of IN. Whether IN contains SIM(s) and their functions are currently under investigation. The most well‑characterized SIM is defined as V/I-x-V/I-V/I or V/I-V/I-x-V/I/L, where x can be any amino acid, in a parallel or anti-parallel orientation [56,57,58]. The examination of the HIV IN aa sequence revealed that IN indeed harbors three putative SIMs: SIM1 72IILV75, SIM2 200IVDI204 and SIM3 257IKVV260. Future studies, including mutagenesis and biochemical analysis of the covalent SUMO conjugation and possible SIM‑mediated non-covalent SUMO binding of IN, are needed for better understanding of the SUMO‑regulated interactions between IN and cofactors and their functions. 

## 4. Acetylation of Lys Residues in the CTD of HIV-1 IN by the Cellular Histone Acetyltransferase (HAT) p300 and GCN5

Like ubiquitination and SUMOylation, acetylation is another PTM that targets Lys residues on substrates. Lysine acetylation has been observed in the histone proteins and nonhistone proteins of both nuclear and cytosolic origin. During acetylation, one single acetyltransferase transfers acetyl groups to either the α-amino group of amino-terminal residues or to the ε-amino group of Lys residues of the substrates [59]. Acetylation is reversed by histone deacetylases (HDACs). Histone acetylation is related to the chromatin structure and transcriptional activity, whereas acetylation of nonhistone proteins regulates protein activity, localization, protein-protein or protein-DNA interactions, and stability/degradation [59,60]. Two HATs, p300 and GCN5, bind and modify IN both* in vitro* and *in vivo* [5,23]. Structurally, both p300/CBP and GCN5 contain a HAT domain and a bromodomain, in which the HAT domain confers acetylation activity, whereas the bromodomain, a conserved protein‑interaction module, selectively targets acetylated Lys residues [61]. While both of these proteins acetylate IN at Lys residues K264, K266, and K273, K258 is exclusively modified by GCN5 (Figure 1) [5,23]. Consistent with this observation, deletion analysis of IN revealed that the C-terminal last 24–44 aa of IN are the binding interface for both HATs (aa 264–288 for p300 and aa 244–288 for GCN5). Moreover, the binding models of IN/p300 and the IN/GCN5 complexes have been solved based on structural studies and experimental mutagenesis in an independent study [62]. It is known that the IN CTD is critical for its DNA binding [63], and acetylation is able to increase DNA affinity through the neutralization of the positive charge of the target Lys residues [64]. Not surprisingly, the acetylation of IN by these two HATs increases the binding affinity of IN/DNA, and other functions of IN acetylation include the positive regulation on IN enzymatic activity and integration [5,23]. However, the importance of IN acetylation was questioned by another study in which HIV-1 virus containing an untagged K(264/266/273)R IN was fully replication competent, and the integration frequency was modestly impaired, even though IN acetylation was confirmed [65]. The discrepancy was explained by the usage of an epitope tag at the C-terminus of IN, adjacent to the acetylation and mutation sites [65]. Nonetheless, the exact functions of IN acetylation, especially in the setting of viral replication, still await further characterization.

To search for cellular proteins selectively interacting with acetylated IN, a yeast two-hybrid system was employed to screen for cellular protein candidates involved in the acetylation of IN [66]. The study identified 13 cellular proteins that specifically bound acetylated IN, including transcriptional factor, chromatin remodeling factor and nuclear import protein, among others [66]. Among these, LEDGF/p75 and Krüppel-associated protein 1 (KAP1 or TRIM28) were listed as potential candidates [66]. A follow-up study by the same group revealed that KAP1 binds and deacetylates IN by recruiting the deacetylase HDAC1, thus reducing integration efficiency [24]. This finding underscored KAP1 as another molecular control of HIV-1 IN acetylation and its related integration. 

## 5. Phosphorylation of HIV-1 IN by the Cellular Kinase JNK in the Ser Residue of Its Core Domain

Phosphorylation is also a reversible PTM, in which kinases phosphorylate protein substrates and phosphatases dephosphorylate proteins, acting as an on/off switch. Phosphorylation predominantly targets protein subjects on Ser, Thr or Tyr residues in eukaryotes [67]. Protein kinases are categorized into two main groups: the Ser/Thr-specific kinases and the Tyr-specific kinases. During phosphorylation, the catalytic subunits within the kinases catalyze the transfer of the terminal γ‑phosphate of ATP to the hydroxyl oxygen of the Ser, Thr or Tyr residue of the substrate [67]. This phosphoryl group replaces neutral hydroxyl groups on Ser, Thr or Tyr with negatively charged phosphates in the modified protein, which can further interfere with or enhance binding of certain proteins. Thus, the reversible phosphorylation/dephosphorylation plays key roles in the control of many distinct cellular processes. 

Phosphorylation of IN was first discussed by Manganaro* et al.* in 2010 [11]. IN has been shown to be phosphorylated at activated T lymphocytes by cellular kinase c-Jun N-terminal kinase (JNK) at Ser 57 (Figure 1) [11]. The modified form of IN is then recognized and stabilized by cellular peptidyl prolyl-isomerase enzyme Pin1, which is required for efficient HIV-1 integration and infection [11]. To understand the possible linkage between the phosphorylation of IN and other identified PTMs, the acetylation and ubiquitination of IN were studied in this same report [11]. The antagonism between phosphorylation and acetylation has been extensively reported. For instance, the acetylation of STAT1 counteracts Interferon-induced STAT1 phosphorylation [68]. However, the results suggested that the phosphorylation and acetylation of IN are two independent events [11]. Interestingly, there is a direct linkage between phosphorylation and the ubiquitination of IN. The observation that the IN mutant S57A, in which phosphorylation is abolished, is more ubiquitinated, suggests that the phosphorylation and ubiquitination of IN antagonize one another in the regulation of IN stability [11]. This raises the question as to how the phosphorylation of IN impacts its stability. Pin1, which is a peptidyl-prolyl *cis/trans* isomerase, binds and catalyzes conformational changes of phosphorylated proline-directed Ser/Thr residues from *cis* isomers to a more stable *trans* configuration in target proteins [26]. Indeed, experimental evidence has confirmed that Pin1 binding to phosphorylated IN leads to a prolonged IN half-life [11]. Such conformational change of IN by Pin1 might result in the inaccessibility of components from the host UPS, such as ubiquitin and E3 ligase, subsequently resulting in more stable IN.

## 6. Conclusions

Like all viruses, the replication of HIV-1 relies heavily on host proteins due to the limited number of viral genome products. A number of proteins and/or cellular pathways are hijacked by HIV-1 to efficiently complete the replication cycle. In addition, viral proteins such as HIV-1 IN and Tat are extensively modified by various PTMs and consequently contribute significantly to viral replication. These modifications are tightly controlled by various cellular proteins as discussed above (also see Table 1 and Figure 2). Furthermore, the PTMs, including ubiquitination, SUMOylation, acetylation and phosphorylation, mingle with each other, either antagonistically or cooperatively, providing another level of cellular control. Among these four major PTMs that IN is subject to, SUMOylation and phosphorylation have only recently been reported and are poorly understood. The important linkages among the network of ubiquitination, acetylation, SUMOylation and phosphorylation are still missing. Meanwhile, the exact functions of various IN PTMs, especially in the setting of viral replication, still await further characterization. Proteome-wide screening would be a useful and important research tool that would facilitate a better understanding of the network interactions between HIV-1 IN and cellular cofactors along with different PTMs. The use of a tethered catalysis yeast two‑hybrid system, which was invented to study protein-protein interactions that require PTMs, would allow for high-throughput experimental screening [69]. For example, using this method, a study was conducted to identify the cellular proteins binding to the acetylated form of IN [66]. In addition, other new technologies, such as tandem affinity purification together with high-accuracy mass spectrometry, also provide useful tools for the study of host-virus protein complexes (reviewed by Ole N. Jensen) [70]. 

In summary, the regulation of HIV-1 IN by PTMs is complicated by various cellular proteins and is a highly relevant and active research area. A better understanding of these virus-host protein-protein complex interactions will certainly lead to exciting findings and potentially may uncover a new intervention target for treating HIV-1 infection. 
